# Initiating head development in mouse embryos: integrating signalling and transcriptional activity

**DOI:** 10.1098/rsob.120030

**Published:** 2012-03

**Authors:** Ruth M. Arkell, Patrick P. L. Tam

**Affiliations:** 1 Early Mammalian Development Laboratory, Research School of Biology, College of Medicine, Biology and Environment, Australian National University, Canberra, Australian Capital Territory, Australia; 2 Embryology Unit, Children's Medical Research Institute, University of Sydney, New South Wales, Australia; 3 Discipline of Medicine, Sydney Medical School, University of Sydney, New South Wales, Australia

**Keywords:** mouse embryo, head formation, signalling, gene transcription, morphogenesis

## Abstract

The generation of an embryonic body plan is the outcome of inductive interactions between the progenitor tissues that underpin their specification, regionalization and morphogenesis. The intercellular signalling activity driving these processes is deployed in a time- and site-specific manner, and the signal strength must be precisely controlled. Receptor and ligand functions are modulated by secreted antagonists to impose a dynamic pattern of globally controlled and locally graded signals onto the tissues of early post-implantation mouse embryo. In response to the WNT, Nodal and Bone Morphogenetic Protein (BMP) signalling cascades, the embryo acquires its body plan, which manifests as differences in the developmental fate of cells located at different positions in the anterior–posterior body axis. The initial formation of the anterior (head) structures in the mouse embryo is critically dependent on the morphogenetic activity emanating from two signalling centres that are juxtaposed with the progenitor tissues of the head. A common property of these centres is that they are the source of antagonistic factors and the hub of transcriptional activities that negatively modulate the function of WNT, Nodal and BMP signalling cascades. These events generate the scaffold of the embryonic head by the early-somite stage of development. Beyond this, additional tissue interactions continue to support the growth, regionalization, differentiation and morphogenesis required for the elaboration of the structure recognizable as the embryonic head.

## 2. Establishing the blueprint of the embryonic head

### 2.1. Prelude to germ layer formation

**Figure 1.**
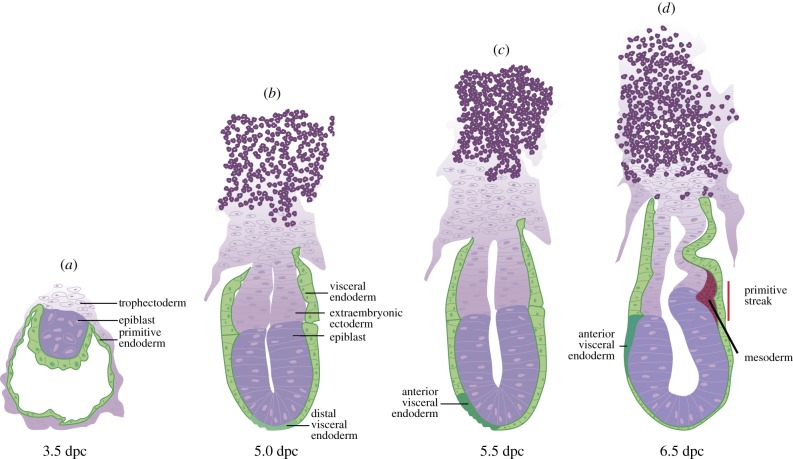
Development of the mouse embryo from 3.5 dpc (days post coitum) to 6.5 dpc. (*a*) Blastocyst containing an inner cell mass comprising the epiblast and the primitive endoderm. (*b,c*) Egg cylinder embryo at 5.0 dpc with distal visceral endoderm, and 5.5 dpc with anterior visceral endoderm. (*d*) Early-streak embryo at 6.5 dpc, with formation of the primitive streak and the nascent mesoderm.

During the initial phase of mouse development, the zygote (fertilized egg) undertakes multiple rounds of cleavage divisions and concurrently allocates cellular progeny to three tissue lineages (trophectoderm, epiblast and primitive endoderm) of the resultant embryo, known as the blastocyst. The blastocyst is built as a vesicular structure (figure 1*a*) with an epithelial layer (the trophectoderm) enclosing a cavity (the blastocoel) and, attached to the wall on one side of the blastocoel, a cluster of cells that constitutes the inner cell mass (ICM). The ICM is further segregated into the epiblast, which gives rise to the entire embryo and some components of the foetal extraembryonic membranes, and the primitive endoderm that lines the luminal surface of the cluster of epiblast cells [1].

Following the implantation of the blastocyst, the epiblast and primitive endoderm grow into the blastocoel to form a cylindrical embryo—the egg cylinder (figure 1*b*). The embryo is composed of a column of three tissues: proximally the extraembryonic ectoderm (derived from the trophectoderm), distally the epiblast (a cup-shaped epithelium derived from the ICM) and, enveloping these two tissues, a thin layer of visceral endoderm (descended from the primitive endoderm). The embryo, while maintaining the cylindrical architecture, continues to grow by cell division, and cells move around in the visceral endoderm and the epiblast (figure 1*c*). Through the process of gastrulation, cells from the epiblast are allocated to three definitive germ layers: the ectoderm, the mesoderm and the endoderm (figure 1*d*). The formation of latter two layers is accomplished by morphogenetic cell movement: ingression of epiblast cells at the site of epithelial–mesenchyme transition (the primitive streak), the organization of the ingressed mesoderm progenitors into a mesenchymal layer and the incorporation of the endoderm progenitors into the pre-existing layer of visceral endoderm [2,3].

### 2.2. The building blocks

**Figure 2.**
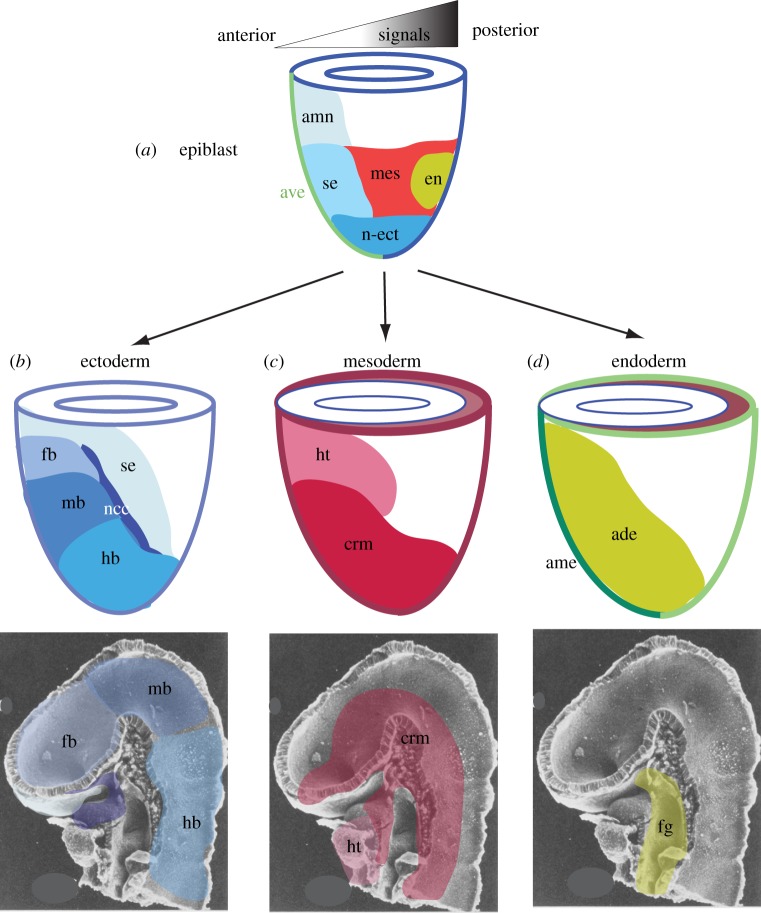
Allocation of the germ layer derivatives to the embryonic head structures. (*a*) Regionalization of germ layer progenitors in the epiblast elicited by the graded signalling activity across the prospective anterior–posterior plane of the embryo. (*b–d*) Allocation of epiblast-derived cells during gastrulation to (*b*) the ectoderm tissues that contribute to the brain, neural crest and the surface ectoderm, (*c*) the mesoderm tissues in the cranial mesenchyme and the heart, and (*d*) endoderm tissues of the embryonic foregut. The fate maps of the progenitor tissues of the embryonic head reveals that the domains and boundaries of the progenitors in the three germ layers are generally aligned with each other, although a clear demarcation of head versus non-head progenitors is not yet evident at the late gastrulation stage. ade, anterior definitive (gut) endoderm; ame, anterior mesendoderm; amn, amnion ectoderm; ave, anterior visceral endoderm; crm, cranial mesoderm; en, endoderm; fb, forebrain; fg, foregut; hb, hindbrain; ht, heart; md, midbrain; mes, mesoderm; ncc, neural crest cells; n-ect, neuroectoderm; se, surface ectoderm.

The emergence and the developmental trajectory of germ layer derivatives have been examined in the mouse embryo extensively by fate-mapping analysis at developmental stages from immediately before the onset of gastrulation to the formative stage of head morphogenesis (figure 2). These studies have identified the location of progenitor cells and their descendants that contribute the tissues that make up the embryonic head. Derivatives of the three germ layers contribute to different parts of the brain, the facial primordia and the upper digestive tract.

#### 2.2.1. Ectoderm

At the pre-gastrulation stage, germ layer progenitors are broadly regionalized in the epiblast: ectoderm in the prospective anterior and distal domains, and endoderm and mesoderm in the prospective posterior domain, with a predominantly mesoderm domain intercalated between these two regions (figure 2*a*). After gastrulation is initiated, the ectoderm progenitors can be resolved into those destined for surface ectoderm (body covering) and neuroectoderm, respectively. Within the neuroectoderm domain, cells that contribute to the brain are localized more anteriorly than those of the spinal cord (figure 2*b*). During gastrulation, this neuroectoderm population expands anteriorly and proximally, and eventually occupies over two-thirds of the area of the ectoderm layer by the time the embryo has formed a complete layer of mesoderm. This is accompanied by an emerging pattern of regionalization of the progenitors for ectoderm tissues of the head. Progenitor cells of the non-neural derivatives (e.g. surface ectoderm and buccal lining) and neural tissues of forebrain, the midbrain and the hindbrain are localized, in the respective anterior–posterior order, to domains that are increasingly farther away from the rostral-most border of the ectoderm (figure 2*b*). In addition, the progenitors of another non-neural ectoderm derivative, the neural crest cells that give rise to the ecto-mesenchyme and cranial ganglia in the head, are mapped to the border region of neural and surface ectoderm domain [4].

When tracking the segmental fate of the neuroectoderm cells in the presumptive forebrain domain, it was noted that while there is a preference of these cells to colonize the forebrain, some descendants are also found in the more posterior brain parts [5]. This tendency of clones expanding to neighbouring brain parts diminishes for neuroectoderm cells of more advanced embryos, suggesting that the neural progenitor cells are either becoming more restricted in their fate or progressively confined to a spatially defined domain during development [6]. The relative size of the domain of progenitors does not correlate with the final size of the brain part. Specifically, the forebrain has undergone a disproportionate expansion during neurulation, which is underscored by the wide area covered by the clones of forebrain neuroectoderm cells. This requirement of tissue growth for morphogenesis underpins the vulnerability of the forebrain in developmental errors that lead to head truncation.

#### 2.2.2. Mesoderm

Cells in the mesoderm domain of the epiblast of embryos at the onset of gastrulation have been shown to contribute to head (cranial) mesoderm and other somatic mesoderm. During germ layer formation, epiblast cells are allocated sequentially to mesoderm of the heart, head and the trunk along the anterior–posterior body axis [7]. After ingression through the primitive streak, the heart and cranial mesoderm progenitors are displaced as a tissue sheet to the anterior region of the embryo and ultimately underlie the prospective brain domains within the ectoderm (figure 2*c*). The cranial mesoderm together with the ectomesenchyme derived from the neural crest cells give rise to the skeleton, muscles, vascular and connective tissue of the head and face [8,9].

The progenitors of another mesoderm population (the axial mesoderm) are co-localized with those of the endoderm. The cells of the axial mesoderm ingress through the anterior segment of the primitive streak and extend along the embryonic midline by convergent extension to reach the entire length of the body axis. The resulting midline structure underlies the brain and spinal cord, and is given different names according to its position in the anterior–posterior axis: the axial mesoderm that underlies the forebrain is the prechordal plate and that which associates with the rest of the brain is the anterior notochord, whereas the segment underneath the spinal cord is the notochord [10]. During its ontogeny, cells of the axial mesoderm are transiently part of the endoderm layer but later separate from it to take up a position among the mesoderm tissues. To distinguish these phases of development, for the period in which the axial mesoderm is contiguous with the flanking endoderm, it is referred to as axial mesendoderm.

#### 2.2.3. Endoderm

In the pre-gastrulation embryo, a layer of endoderm cells (the visceral endoderm) is already present. Contrary to the conventional concept that the visceral endoderm gives rise only to the non-embryonic tissue that lines the extraembryonic yolk sac, its descendants can contribute to the anterior and posterior segments of the embryonic gut [11]. The ultimate fate of these cells in the digestive tract is not known. The bulk of gut (definitive) endoderm cells is recruited from their progenitors in the epiblast (figure 2*a*). Definitive endoderm ingressed at the anterior segment of the primitive streak is incorporated into the pre-existing visceral endoderm by intercalation over a wide area and not restricted to the sites in the immediate vicinity of the primitive streak. Through a concerted movement, cells destined for the upper digestive tract (the foregut) congregate to the anterior region of the endoderm layer (the anterior definitive endoderm, figure 2*d*) underlying the cranial and heart mesoderm and the prospective brain domains in the ectoderm [12]. During head morphogenesis, the endoderm forms the lining of the embryonic foregut and the associated organs [13–15].

The formation of the three germ layer derivatives henceforth completes the building blocks of the head. The ensuing morphogenetic movement that brings these tissue components to their proper place in the body plan establishes the blueprint of the embryonic head by the early-somite stage of development. Later events will continue to build upon this scaffold until the fully differentiated head structures emerge.

## 3. Anterior–posterior polarity and signalling centre in the anterior visceral endoderm

### 3.1. Proximal–distal regionalization of gene activity in the egg cylinder

Analysis of gene expression in the egg cylinder embryo has revealed that the transcripts encoding components of signalling pathways such as that of Nodal, BMP and WNT are localized to specific tissue compartments [3,16–19]. For example, signalling ligand genes such as *Bmp2*, *Bmp8b, Bmp4, Wnt2b, Wnt3*, and activating convertase enzymes for Nodal such as *Furin* and *Pcsk6* are expressed in the extraembryonic ectoderm or the proximal population of visceral endoderm. In contrast, factors that antagonize the TGF-beta and WNT signalling activity, such as *Cerl, Lefty1* and *Dkk1*, are expressed in the distal population of the visceral endoderm (the distal visceral endoderm, DVE). In the epiblast, *Nodal* is expressed in the proximal domain whereas the *Cripto* receptor is uniformly expressed. Notwithstanding the caveat that gene expression domains may not reflect the range of action of the signalling factors, the regionalization of transcripts points to a graded pattern of high to low signalling activity in the proximal–distal dimension of the egg cylinder.

### 3.2. Ontogeny of distal visceral endoderm and anterior visceral endoderm

By tracing the trajectory of *Lefty1*-expressing cells that first emerge in the blastocyst, the DVE cells are found to descend from a subset of primitive endoderm cells [20,21]. It remains unclear how these progenitors and their progeny are translocated en masse from a lopsided position in the primitive endoderm to the distal site in the visceral endoderm over a 2-day period of development. A possible mechanism is that the displacement of these *Lefty1*-active cells is driven by their response to Nodal and WNT signals such that they are compelled to move away from regions of high signal activity and congregate to the distal part of the visceral endoderm. Subsequently, the population of *Lefty1*- and *Cer1*-positive cells expands, and later these cells are relocated to anterior region of the visceral endoderm (and become known as the anterior visceral endoderm, AVE) [22]. Contrary to the notion that these AVE cells are descendants of the DVE cells, recent lineage analysis reveals that they are of separate lineages. The DVE cells do not give rise to AVE cells, although they share similar molecular properties, intermingle with the AVE cells and participate in similar act of cell movement to the anterior side of the embryo [20]. The progenitors of the AVE are generated *de novo* from other visceral endoderm. This is likely to be accomplished via the modulation of BMP inductive activity [23–25], but does not require the presence of DVE cells [20].

### 3.3. Acquisition of anterior–posterior body axis polarity

Both the DVE cells and AVE progenitors are localized initially to the distal sites of the egg cylinder. In this position, the antagonistic activity emanated from these cells may contribute to the alignment of a signalling axis in the proximal–distal plane of the embryo. By transforming the cup-shaped epiblast and the associated visceral endoderm to a flat disc-like configuration, it can be visualized that the signal activity may lead to a radially symmetrical body plan [26]. The breaking of this radial symmetry may be achieved by localizing the source of signals or that of the antagonists to one side of the embryo and thereby creating an asymmetry of the body plan. The movement of the mixed populations of *Lefty1* and *Cer1*-expressing DVE and AVE cells to the prospective anterior pole of the embryo is therefore key to the acquisition of the anterior–posterior polarity by the embryo.

While the presence of the DVE is not a prerequisite for the *de novo* formation of AVE cells, DVE cells are required for the anterior displacement of the AVE cells [20]. Visceral endoderm cells that are recruited to the AVE and begin to express *Lefty1* join the anterior stream of cells. Whether the DVE cells act to initiate as well as to guide the movement of the AVE cells and the mechanistic basis for such navigational activity are not known. Likewise, the morphogenetic forces that drive the directional movement of the visceral endoderm cells are not fully known. Experimental manipulations of Nodal/Lefty1 and WNT/Dkk1 signalling activity reveal that the visceral endoderm cells respond to differences in signal intensity (by travelling towards regions of low signal activity) [27,28], and to the differential proliferative activity of the epiblast [29]. Loss of *Otx2* function, which is accompanied by the loss of *Dkk1* activity, impairs the anterior movement of the visceral endoderm [30]. Enforced expression of *Dkk1* under the control of Otx2 can restore the migratory activity of the *Otx2*-deficient cells [27]. These experiments provide circumstantial evidence that expression of Nodal and WNT antagonists by the AVE cells influences their migratory behaviour. *Lefty1*-positive AVE cells remain in the anterior domain of the endoderm, whereas DVE cells that lose *Lefty1* activity after they reach the anterior site continue to migrate but follow a different path to the lateral region of the embryo.

### 3.4. Regionalization of signalling activity and impact on epiblast patterning

The displacement and expansion of the DVE and AVE cells to the anterior side of the embryo establish an anterior source of antagonistic activity against Nodal and WNT signals. Concurrently, the expression domain of *Nodal* and *Wnt3* retreats to the posterior side of the embryo. The proximal–distal signalling axis is consequently realigned to the prospective anterior–posterior body axis of the embryo. Specifically for the WNT signalling pathway, other antagonists in addition to *Dkk1* (e.g. *Sfrp1*, *Sfrp5*, *Cer1*) are expressed in the anterior part of the embryo, whereas WNT ligands (such as *Wnt3*, *Wnt2b* and *Wnt8a*) are expressed in the posterior part of the embryo [16,31]. These opposing domains of antagonists and ligands presumably establish a low to high gradient of WNT signalling activity across the anterior–posterior plane of the embryo. Similarly, a Lefty1–Nodal and Cer1–BMP signalling gradient may also be established. The provision by the AVE of secreted inhibitors such as Dkk1, Cer1 and Lefty1 to modulate WNT, BMP and Nodal factors is critical for the differentiation of the epiblast. The combined loss of function of *Cer1* and *Lefty1* leads to the formation of an enlarged primitive streak (i.e. enhanced specification of mesoderm and endoderm lineages). This phenotype is partly suppressed when Nodal signalling is decreased, indicating that these molecules normally constrain the level of Nodal signal within the epiblast [32]. Likewise, an inability to establish the AVE (for example, in *Otx2* mutants) results in ectopic expression of mesoderm markers in the epiblast, a manifestation of the posteriorization of the epiblast [27].

It may be noted that the area traversed by the migrating AVE and final residence of the AVE match the domain of the ectoderm progenitors (amniotic, surface and neural ectoderm) in the epiblast. In contrast, the epiblast in the domain of high WNT and Nodal activity is destined for the formation of the mesoderm and endoderm (figure 2). A crucial role of the AVE is therefore to maintain the naive characteristics of the anterior epiblast and to prevent inappropriate differentiation to non-ectodermal cells. Recently, evidence has emerged that the AVE is also a source of instructive signals. *Bmp2* is expressed in the AVE of the early gastrula, and then in the node, anterior definitive endoderm and prechordal plate of the late gastrula. Conditional ablation of visceral-endoderm-derived *Bmp2* rescues some, but not all, of the *Bmp2*-null phenotypes: anterior definitive endoderm and prechordal plate are specified, but the development of head and foregut is perturbed [33]. Apparently, the signalling activity of the AVE has a lasting impact on the differentiation and morphogenesis of epiblast-derived tissues into head structures. However, tissue transplantation experiments have revealed that AVE itself is not sufficient for inducing or maintaining the differentiation of the epiblast into anterior neural tissues [34], suggesting that the AVE may act primarily as a source of permissive signals for the development of anterior structures.

## 4. Gastrulation and anterior midline signalling

### 4.1. Sources of morphogenetic activity

Lineage-tracing studies have revealed that during gastrulation, descendants of the visceral endoderm remain among the anterior definitive endoderm, the gastrula organizer (node) and the anterior mesendoderm [11]. This raises the possibility that some AVE descendants may persist throughout gastrulation and continue to perform the AVE-related morphogenetic function. However, the domain previously occupied by the AVE is mostly populated by the incoming anterior definitive endoderm (ADE) and the axial mesendoderm (AME). Both tissues are recruited from cells that ingress through the anterior segment of the primitive streak that encompasses the gastrula organizer. These tissues reach the anterior region of the embryo by separate morphogenetic tissue movement along the midline and the lateral regions underneath the cephalic neural primordial [10]. Similar to the AVE, the ADE and the AME are the source of antagonistic activity to WNT and BMP signalling, and they express *Dkk1*, *Chrd* and *Noggin*.

**Figure 3.**
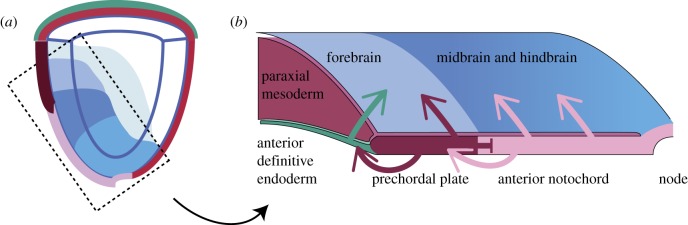
Inductive interaction between the germ layer derivatives during head formation. (*a*) A schematic diagram of the right-hand half of the late-streak embryo showing the domains of brain progenitors in the ectoderm and the opposing paraxial mesoderm, endoderm and the axial mesendoderm (prechordal plate and anterior notochord, derived from the node). The boxed area of (*a*) is shown in (*b*), which depicts the planar (inductive and suppressive) interaction between the prechordal plate and the anterior notochord, the induction by the prechordal plate to maintain the anterior definitive endoderm, and the vertical (i.e. between germ layers) induction of the neural primordium by the endoderm and the axial mesendoderm.

The critical role of ADE and AME in promoting anterior patterning is revealed by the truncation and malformation of head structures when ADE and/or AME development or function is perturbed. A failure of differentiation or disruption of tissue movement (e.g. in *Mixl1, Lhx1, Foxa2* and *Zic2* mutants), or the loss of morphogenetic signalling activity (e.g. *Shh, Dkk1, Chrd* and *Nog*), impairs early stages of head formation [35–41]. The AME is composed of the prechordal plate and the anterior notochord, which functionally interact with each other via planar signals. For example, the prechordal plate is not maintained in the absence of the anterior notochord [41,42], and the prechordal plate is required to suppress the ectopic activation of *Gsc* in the anterior notochord [42]. Furthermore, the prechordal plate provides inductive activity for sustaining the differentiation of the ADE [37]. The identity of the molecules that direct the progressive differentiation of the AME and ADE is not known, but the intricate network of cross-talk among these three epiblast-derived tissues is central to the maintenance of the anterior neural characteristics of the neuroectoderm and the formation of the head structures (figure 3).

### 4.2. Balancing the signalling activity

**Figure 4.**
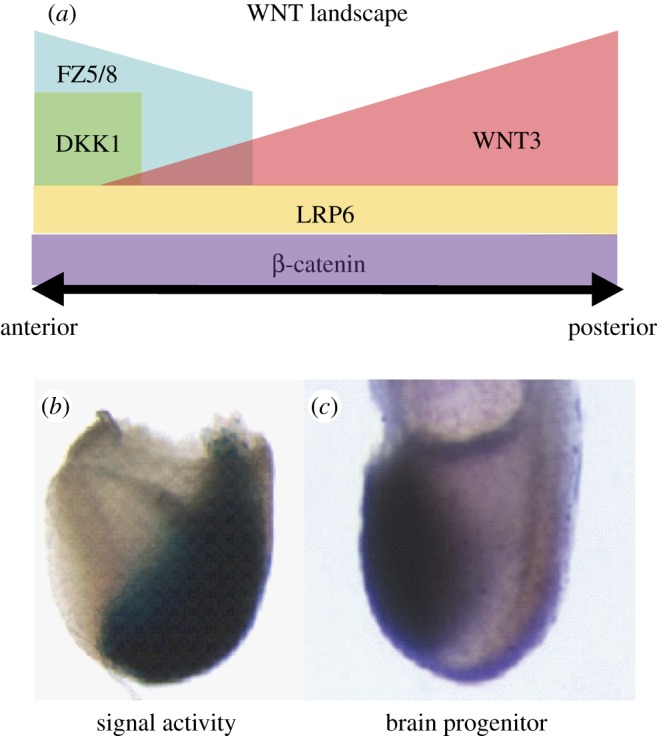
Specification of the brain progenitors is facilitated by WNT signalling activity. (*a*) The regionalized activity of signalling components sets up a signalling landscape with (*b*) reduced WNT signal activity (low reporter expression) in (*c*) the domain of brain progenitor (marked by *Otx2* expression). Source of figures: (*a*) fig. 9, Fossat *et al*. [44]; (*b*) fig. 1c, Lewis *et al*. [43] (permission for use by authors under copyright agreement with Development, Company of Biologists Ltd).

Morphogenetic signals must be delivered at the right time, place and strength to elicit proper lineage differentiation and morphogenesis of the head progenitor tissues. The complex mechanisms that localize, constrain and refine Nodal signals at the onset of gastrulation have been reviewed elsewhere [17]. Similarly, complex mechanisms are employed to balance the WNT signals that permit the early events of head formation [43]. In the embryo at gastrulation, the expression domain of *Wnt3* in the posterior region juxtaposes that of *Dkk1*, which separates the *Wnt3* domain from the *Fzd8* receptor domain in the anterior region. The *Dkk1* and *Fzd8* domains together shadow the domain of brain and cranial mesoderm progenitors in the ectoderm and mesoderm, respectively (figure 4). These molecular annotations of the fate map point to a plausible scenario in which Wnt3 signal emanating from the posterior epiblast and the primitive streak is dampened by the Dkk1 antagonist such that a reduced level of signalling activity is perceived by the receptive head progenitor tissues. While other WNT antagonists are expressed at this stage of embryonic development, the loss of *Dkk1* alone can cause a major disruption of head development. This finding suggests that the function of Dkk1 cannot be replaced by other antagonists, which display no changes in their expression in the *Dkk1*-null mutant embryo. When Wnt3 activity is reduced (by genetically silencing one *Wnt3* allele) on the *Dkk1*-null background, head development is partially restored. This indicates that the primary target of Dkk1 is the Wnt3-mediated signalling cascade and that other WNT factors (which do not change their expression significantly in the *Wnt3* and *Dkk1* mutants) might play a lesser role in head development [43].

Wnt3 is a canonical WNT signalling molecule and these experiments therefore also imply that Dkk1 exerts its influence by modulation of canonical WNT signalling [44]. This has been confirmed by the demonstration that different permutations of mutations of the antagonist (*Dkk1*), the co-receptor (*Lrp6*) and transcription co-activator (*β*-*catenin*) produce phenocopies of the head defects associated with excessive canonical signalling activity. Furthermore, the different degrees of elevation of WNT signalling activity caused by the three mutated genes correlate with the severity of the head defects, with the tissues of the anterior brain region being more sensitive to changes in the signalling activity than those of the posterior regions. Therefore, Dkk1 acts by controlling the level of canonical signalling activity perceived by the target tissue, and a stringent control of the signal strength at different locations in the anterior–posterior plane is critical for the development of specific brain parts. *Wnt3* is expressed in a relatively narrow window of embryonic development, first in the proximal visceral endoderm and then progressively confined to the posterior visceral endoderm, and activated in the posterior epiblast and the early primitive streak. The requirement for Dkk1 modulation of Wnt3 signals is therefore confined mainly to pre- and early gastrulation.

### 4.3. Intersection with transcriptional activity

The WNT antagonist Dkk1 has been shown to be a direct transcriptional target of the WNT/β-catenin-dependent activity [45,46]. The downstream activation of an antagonist thereby provides a negative feedback mechanism for the modulation, rather than wholesale inhibition, of the canonical signalling activity. This feedback mechanism is disrupted when one allele of both *Dkk1* and *Wnt3* is ablated. The reduced WNT signal acting on only one functional *Dkk1* allele leads to a decreased amount of antagonist that is inadequate to modulate the Wnt3 activity [43]. Negative modulation of signalling activity is also achieved at the level of transcription of Wnt pathway components. For example, *Gsc* activity in the prechordal plate, which negatively regulates itself, represses the transcription of WNT ligands (such as *Wnt8* [47]), and *Sox17* in the ADE may downregulate the expression of β-catenin target genes through the physical interaction and redeployment of β-catenin for other non-signalling cellular functions [48,49].

**Figure 5.**
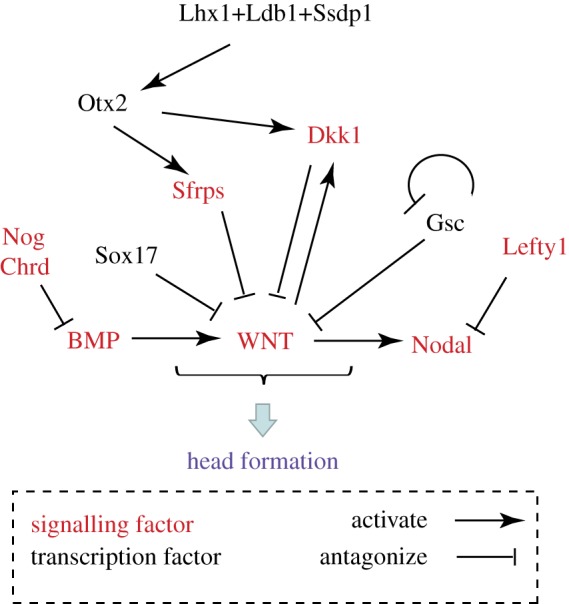
The intersection of signalling and transcriptional activity culminates in the suppression of BMP, WNT and Nodal signalling for head formation. Sources of signals in the gastrulation stage embryo: Bone Morphogenetic Protein (BMP) from the extraembryonic ectoderm and the posterior epiblast, and WNT (Wnt2b, Wnt3, Wnt3a and Wnt8a) and Nodal from the posterior epiblast and primitive streak. BMP antagonists Noggin (Nog) and Chordin (Chrd); Nodal antagonist Lefty1; WNT antagonists Dickkorf-1 (Dkk1) and Secreted frizzled-related proteins (Sfrps). Transcription factors: Goosecoid (Gsc), LIM homeobox protein 1 (Lhx1), LIM domain binding 1 (Ldb1), Single-stranded DNA binding protein 1 (Ssdp1); Orthodenticle homologue 2 (Otx2), SRY-box containing gene 17 (Sox17).

Similar to the phenotypic effect of loss of *Dkk1* function, mutations of two transcription factor genes, *Lhx1* and *Otx2*, produce head truncation defects [50,51]. Loss of *Lhx1* is accompanied by the failure to form the AME, through its downstream effect on the expression of non-canonical WNT signalling factors that influence morphogenetic cell movement in the mesoderm and the AME [39]. Loss of *Lhx1* function also elicits a more global response of the upregulation of WNT response genes and, concurrently, the downregulation of WNT antagonists and also the *Otx2* transcription factor gene (N. Fossat & P. P. L. Tam 2012, unpublished data). Combinations of mutations of *Dkk1, Lhx1* and *Otx2* are associated with the manifestation of head truncation phenotype, albeit in varying degrees with different permutations. While these findings indicate a potential intersection of WNT signalling activity with the transcription of head-forming genes, the underpinning molecular mechanism is not fully known. However, Lhx1 factor is a component of the transcription complex containing Ssdp1 and Ldb1. This complex may be targeted to or cooperating with the *Otx2* gene, which in turn regulates the expression of several WNT antagonists. Loss of *Ssdp1* and *Ldb1* function individually has been shown to cause head defects and reduced expression of antagonists including *Dkk1* in the prechordal plate and Sfrps in the ADE, and combinations of *Ssdp1* and *Lhx1* or *Ldb1* mutant alleles produce phenocopies of head defects [52,53]. These data provide compelling evidence of a functional intersection of transcription activity with the molecular cascade of WNT signalling that promotes head morphogenesis (figure 5). It appears that a complex network of secreted antagonists, co-receptors and transcriptional feedback mechanisms regulate the time, space and strength of the WNT signals that drive the initial differentiation and morphogenesis of the progenitor tissues of the murine embryonic head. A landscape of graded WNT signalling activity along the anterior–posterior axis of early embryos is found in a multitude of vertebrate and invertebrate species [54,55]. Similarly, modulating BMP signalling by the antagonist (e.g. Cerberus) emanating from the endoderm is required for anterior patterning of Xenopus embryo [55,56]. The stringent regulation and regionalization of signalling activity may therefore be a highly conserved molecular mechanism of embryonic patterning.

## 5. Conclusion

Our current understanding indicates that the initial events in formation of the murine head rely on graded signalling activity of the WNT, Nodal and BMP pathways. Prior to gastrulation, these signalling cascades together elicit the first overt sign of anterior–posterior polarity when a mixed population of DVE and AVE cells move to the prospective anterior pole of the embryo and form the AVE signalling centre. This centre secretes WNT, Nodal and BMP antagonists, and delimits a region of embryo in which the future neuroectoderm can escape the signals that drive epiblast ingression and differentiation into the definitive endoderm and mesoderm at the primitive streak. The mesoderm and endoderm derivatives (ADE and AME) of the anterior primitive streak replace much of the pre-gastrula visceral endoderm and, like the AVE, they also supply WNT, Nodal and BMP antagonists. Interactions between these definitive tissues generate anterior–posterior differences within the ADE and AME, which maintain the neural character of (and perhaps begin to regionalize) the overlying neuroectoderm. The WNT, Nodal and BMP antagonism provided by these signalling centres is essential for the anterior patterning of the germ layer derivatives and thereby establishing a blueprint of the embryonic head. It stands to reason that, as well as controlling the expression of secreted antagonists, transcription factors expressed at the signalling centres also repress the expression of ligands and the downstream effectors of WNT, Nodal and BMP signalling. The integration of signalling and transcriptional activity in the signalling centres and the progenitor tissues is therefore instrumental for initiating and orchestrating the development of the embryonic head.

## Author profiles

**Patrick Tam** is the Deputy Director and Head of the Embryology Research Unit at the Children's Medical Research Institute. He is a Senior Principal Research Fellow of the National Health and Medical Research Council of Australia, and holds a conjoint appointment as Professor in the Discipline of Medicine at the Sydney Medical School, The University of Sydney. He is also the Mok Hing-Yiu Distinguished Visiting Professor of the Li Ka Shing Faculty of Medicine at the University of Hong Kong.

Patrick's research focuses on the elucidation of the cellular and molecular mechanisms of body patterning during embryonic development. Major effort is aimed at the understanding of the integration of the molecular control of gene activity and the signalling activity that mediates cell–cell communication and the coordination of cell differentiation in the formation of organs and major body parts.

Patrick Tam is an Editor of Development and member of the editorial board of several journals on developmental biology, including Developmental Cell and Developmental Biology, and serves on the scientific advisory board of several major research institutes, including the RIKEN Centre of Developmental Biology, Genome Institute of Singapore and Max Planck Institute of Molecular Genetics. Patrick was awarded the Symington Memorial Prize of the Anatomical Society of Great Britain and Ireland (1987), Dr S. T. Huang-Chan Memorial Medal of Hong Kong University (2003) and the President's Medal of the Australian and New Zealand Society of Cell and Developmental Biology (2007); and elected Fellow of the Australian Academy of Science (2008), Society of Biology (2009) and the Royal Society of London (2011).

**Ruth Arkell** currently heads the Early Mammalian Development Laboratory. She completed her undergraduate studies at the University of Sydney (BSc Hons) and commenced doctoral studies in Mouse Molecular Embryology in 1992 at Edinburgh University. These studies were completed at the National Institute for Medical Research in London in 1996. In order to capitalize on the new opportunities which would arise once the mouse genome sequence was available, she pursued post-doctoral research training (in London and Oxfordshire) in mouse functional genetics. In 2000, she was awarded a UK MRC Career Development Award and established a research group in Oxfordshire to study gene function during mouse gastrulation. In 2006, she was a Sylvia and Charles Viertel Senior Medical Fellowship recipient and moved her research to The Australian National University. In 2009, she was the recipient of the ANZSCDB Young Investigator Award. She continues to use embryology and functional genetics to study the molecular and embryological basis of congenital defects that have their aetiology in gastrulation.
